# Good results in the treatment of ossicular lesions without reconstruction – our explanation for seven cases^☆^

**DOI:** 10.1016/j.bjorl.2016.03.003

**Published:** 2016-04-22

**Authors:** Silvio da Silva Caldas Neto, Mariana de Carvalho Leal, Nelson Caldas

**Affiliations:** Universidade Federal de Pernambuco (UFPE), Departamento de Otorrinolaringologia, Recife, PE, Brazil

## Introduction

The ossicular chain function is well known since mid-19th century. Its presence promotes a sound amplification of about 30 dB. Therefore, one can expect its interruption results in air-bone gap of about 30 dB or more. This discontinuity, represented mainly by erosion of the incus, is commonly encountered in chronic otitis media or, more rarely, in middle ear trauma, either as a consequence of the disease itself or produced by surgical procedures. In many instances, in order to improve or preserve hearing, ossicular reconstruction is part of the treatment, performed using implants of different kinds, usually rigid, to assure an efficient transmission of acoustic energy from the tympanic membrane (TM) to the oval window. However, some cases challenge the common sense about the physiology of the tympano-ossicular unit. The authors present seven patients with ossicular interruption, which, surprisingly, didn’t require a surgical reconstruction to reach a quite good (sometimes excellent) functional result, and further propose an explanation based on acoustic physics knowledge for such cases.

## Case reports

In all patients, the ossicular defect was type A of Austin.^1^ All of them were treated with a canal wall up tympanomastoidectomy, and in all of them a fibrous bridge was noted ligating the malleus or the TM to the stapes. Audiometric data were expressed by the mean air-bone gap at frequencies 500, 1000, 2000 and 4000 Hz. In none of these cases there was any connection between the stapes and the TM other than the fibrous bridge and no tympanic retraction was noticed over the stapes. Clinical, surgical and audiological data from the seven cases are in Table 1.

**Table 1:** Clinical, surgical and audiological data from the seven cases.

Case	Clinical data	Thresholds provided by the fibrous bridge (0,5, 1, 2, 4 kHz)
1	Male, 9 y-o, left cholesteatoma, no hearing complaint. Malleus head and incus absent. Fibrous bridge between the TM and the stapes head. Postoperative follow-up: normal TM. Normal contralateral ear and hearing.	BC: 15 – 20 – 15 – 20
		AC: 15 – 20 – 15 – 20
		Mean gap: 0 dB

2	29 y-o man, left attical cholesteatoma, Bilateral hipoacusis. Incus and malleus head absent. Fibrous adhesion between the TM and stapes head. Postoperative normal TM. Contralateral ear: mild mixed hearing loss.	BC: 00 – 00 – 05 – 20
		AC: 25 – 30 – 25 – 30
		Mean gap: 21.25 dB

3	22 y-o woman; 30 days history of horse fall and immediate facial paralysis. No hearing complaint. Normal TM. Incus completely displaced in the mastoid antrum. Fibrous adhesion between the malleus and stapes head. Normal contralateral otoscopy and hearing.	BC: 10 – 10 – 10 – 10
		AC: 15 – 10 – 20 – 20
		Mean gap: 6.25 dB

4	35 y-o man, right cholesteatoma, slight hipoacusis. Malleus head and incus absent. Fibrous bridge connecting malleus handle to stapes. Postoperative normal TM. Normal contralateral ear.	BC: 20 – 15 – 20 – 20
		AC: 40 – 40 – 35 – 40
		Mean gap: 20 dB

5	33 y-o man; right cholesteatoma, no auditory complaint. Incus absent. A thin filiform fibrous adhesion linked the stapes to the malleus. Post-operative normal TM. Normal left ear.	BC: 05 – 05 – 10 – 10
		AC: 20 – 20 – 25 – 20
		Mean gap: 13.75 dB

6	23 y-o man, left cholesteatoma, no hearing complaint. Malleus head absent. Incus partially absent (lenticular process remained). Fibrous link from the TM to the lenticular process. Postoperative normal TM. Normal contralateral ear.	BC: 10 – 10 – 30 – 50
		AC: 10 – 15 – 30 – 50
		Mean gap: 1.25 dB

7	31 y-o male, right attical cholesteatoma, mild hipoacusis. Complete absence of the incus. Only a connective tissue cord from the malleus handle to stapes head. Normal contralateral ear.	BC: 05 – 10 – 15 – 15
		AC: 20 – 25 – 45 – 40
		Mean gap: 21.25 dB

AC, air conduction; BC, bone conduction.

### Case 1

**Figure 1:**
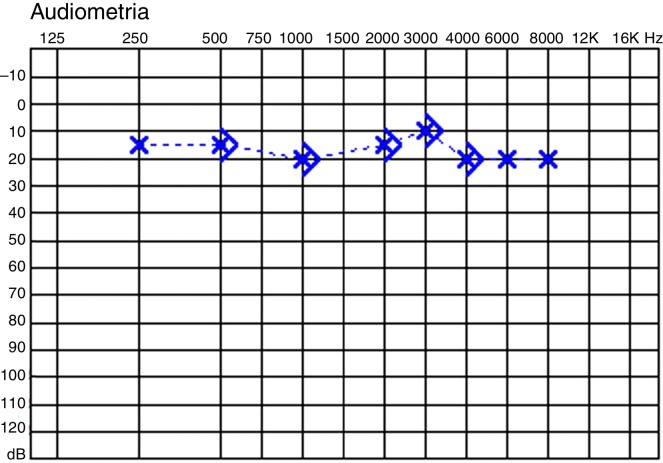
Audiograms of case 1.

Nine-year-old boy, left cholesteatoma, normal hearing. Malleus head and incus removed. Functional reconstruction postponed. Postoperative audiogram: completely closed gap (Fig. 1). During the second examination, fibrous bridge encountered and preserved between the TM and the stapes head. The postoperative follow-up was adequate, with an eventual normal TM and the final audiogram was similar.

### Case 2

29-Year-old man, left attic cholesteatoma, bilateral mixed hearing loss. Incus and malleus head sacrificed. No reconstruction performed. Postoperative audiogram with a mean gap of about 21 dB. During the revision, no residual disease noted. A fibrous adhesion was seen between the TM and the stapes head. Adhesion removed and replaced by cortical bone autograft. Final mean gap: 35 dB.

### Case 3

**Figure 2:**
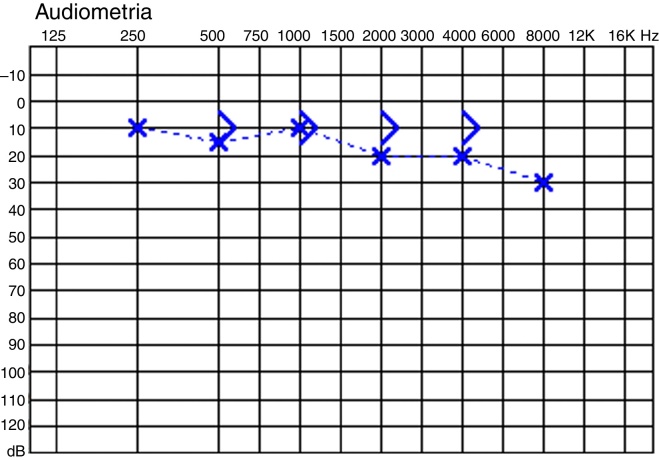
Audiogram of case 3.

22-Year-old woman; 30 days history of horse fall followed by immediate facial asymmetry. House-Brackmann grade VI left facial paralysis. Audiogram: about 6 dB gap (Fig. 2). Transmastoid facial nerve decompression performed. Incus completely displaced in the mastoid antrum. Fibrous adhesion between the malleus and stapes head. One could notice that, pulling the malleus handle outwards, the stapes moved laterally, but it remained stable when the malleus was pushed medially. The adhesion was preserved. No long-term follow-up for this patient.

### Case 4

35-Year-old man, right cholesteatoma, slight conductive hearing loss. Malleus head and incus removed. No reconstruction performed. Postoperative mean gap: 20 dB. On second look, again a fibrous bridge found to connect the malleus handle to the stapes. It was removed and a hydroxy-apatite prosthesis was interposed. Final audiogram: no change on mean air-bone gap.

### Case 5

**Figure 3:**
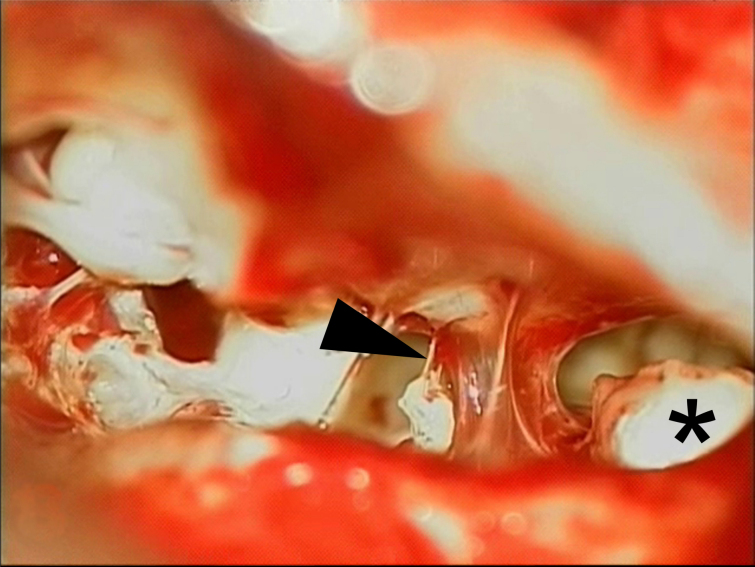
Photograph of case 5 with posterior tympanotomy view of the middle ear. The arrow points to the fibrous bridge linking the malleus to the stapes head. A residual cholesteatoma is seen on right (*).

33-Year-old man, right cholesteatoma, normal auditory thresholds. Incus removed. No reconstruction performed. Postoperative gap of only 15 dB. On revision surgery, a thin filiform fibrous adhesion linked the stapes to the malleus (Fig. 3). Adhesion preserved. No other additional reconstructive maneuver. Similar hearing on postoperative audiometry.

### Case 6

**Figure 4:**
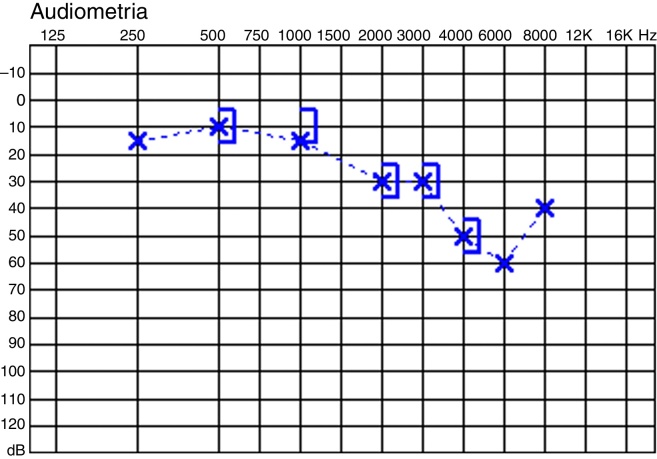
Audiogram of case 6.

23-Year-old man, left cholesteatoma, bilateral normal hearing. Malleus head removed. Incus partially removed, with a small segment of the long process left attached to the stapes due to an extremely fixed incudostapedial joint. Postoperative audiogram: almost no gap (Fig. 4). High frequencies worsening. On second stage, a fibrous link existed from the TM to the remaining tip of the long process. The lateralization of the malleus handle caused a notorious round window reflex, but, when forcing that medially, there was no movement. Bridge preserved. Final audiogram: no gap.

### Case 7

31-Year-old male, right attical cholesteatoma, mixed hearing loss, mean gap of 21 dB. Complete absence of the incus noticed. Only a connective tissue cord from the malleus handle to stapes head, left in place. No postoperative audiogram.

## Discussion

These cases are testimonies of the surprising capacity of the human body to adapt to adverse conditions, with the healing process serving not only to restore the anatomy of an organ or tissue, but also its function. Of course, in most cases, these possibilities cannot be completely reached, but one must be aware to this potential and not be excessively attached to the routine of the surgical technique. The absence of the incus, in a simplistic analysis, could not allow a good sound conduction from the TM to the oval window, unless there was a tympanic retraction over the stapes head, setting up a myringostapediopexy. However, in none of these cases, did we identify any tympanic retraction that could be responsible for the sound transmission to the stapes. It seems clear that, in each one of these patients, the fibrous bridge was the only sound wave conductor. All cases, particularly 1, 3, 5 and 6, achieved functional results that are surprising even when compared with the best series of conventional ossicular reconstruction.^2^, ^3^, ^4^, ^5^ In such cases, the surgeon could question the reliability of the auditory test performed and be tempted to replace the bridge by a regular prosthesis, naturally considered, for its rigidity, more efficacious in sound transmission than a soft structure such as a fibrous adhesion. However, in cases 2 and 4, this replacement was clearly unsuccessful. In three cases (2, 4 and 7), the air-bone gap promoted by the fibrous link was equal or little more than 20 dB. This gap denotes an obstacle to sound transmission, but yet it is clearly far from the expected for an incus absence situation!

How can we explain such good results? Cases 3 and 5 may help us to elucidate the enigma. On them, we mobilized the malleus and observed the effect of this on the stapes or the round window. As could be expected, when the malleus was medialized, there was no movement of those structures because the fibrous bridge, being soft and flexible, deformed without causing any effect on the stapes. However, the lateral traction caused a clear response.

**Figure 5:**
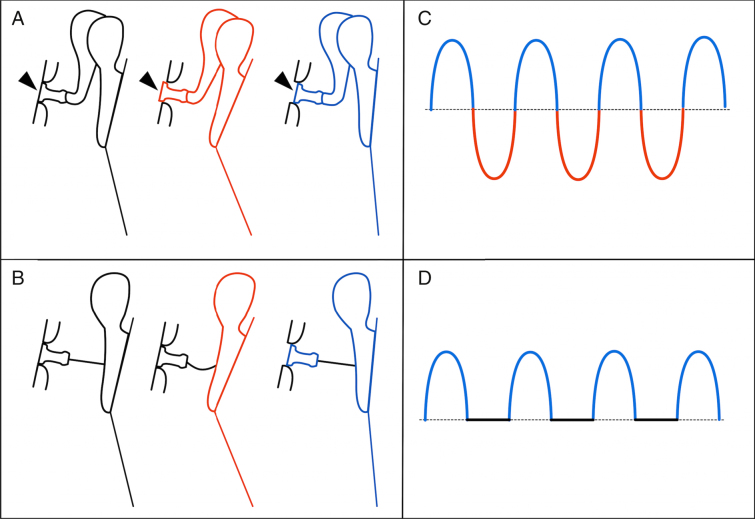
Schematic drawing demonstrating the tympano-ossicular unit movement in normal situation (A) and with the fibrous bridge (B). The black color represents the rest (neutral) position, the compression phase is represented in red, and the rarefaction phase, in blue. Note, on A, the normal behavior of the stapes on oval window (arrowheads), penetrating into the vestibule during the compression (red), and coming out of it during the rarefaction (blue). On B, only the rarefaction produces a stapes movement. On right (C and D), the graphical representation of the resulting perilymph wave is depicted. Note that, in D, only the rarefaction phase is present.

In a normal situation, the TM, stimulated by sound, produces vibratory movements that start with a medialization correspondent to the compression phase of the wave, followed by a return passing through the neutral position, until it reaches a lateral situation (rarefaction phase). These movements repeat continuously while the stimulus lasts. The ossicles follow this movement, with the footplate moving into and out of the vestibule successively. To explain the reported cases, we propose a model which, although not perfect, seems to be sufficient to generate a near normal cochlear stimulus. According to this model, the cycle previously described suffers an amputation of the phase that corresponds to the entrance of the stapes into the vestibule. When the TM medializes, the fibrous bridge deforms and the stapes remains stable. However, when the TM is displaced outwards, the bridge pulls the stapes (Fig. 5). Further, despite this amputation, a stapes periodic oscillatory movement is created, originating a perilymphatic traveling wave. So, the conditions for the vibration of the basilar membrane and stimulation of the sensory organ are established. Obviously, the amputation of the wave determines lower amplitude of the stimulus and that could explain a residual gap, as in cases 2, 4 and 7. But, as already stated, even in these cases, the results were much better than one could anticipate knowing that the incus was absent.

We can find, in the past, evidences of the existence of this effect. Sheehy,^6^ in 1965, had proposed, in cases of absence of the entire ossicular chain, to fill the whole tympanic cavity except the stapes footplate area with Gelfoam^®^, which he filled with a tape of fascia, so to create and maintain, in a long-term basis, a fibrous columella between the footplate and the TM. The author reported a mean gap of 20 dB or less in 40% of cases. Obviously many latter series reported better results employing several implants,^7^, ^8^, ^9^, ^10^ but it is still surprising that, in some cases, a fibrous element could substitute reasonably for the entire ossicular chain. Also curious is the absence of discussions about how it could be obtained with a tissue without any stiffness. We are quite convinced that the explanation is on the model described above.

Of course we are not suggesting that we abandon the ossicular reconstructions. But we must be mindful of the real possibility of this kind of situation and do not ever underestimate the power of these fibrous bridges in maintaining a good sound conduction. And we strongly recommend the preservation of these adhesions in cases where the air-bone gap does not reach 25 dB.

## Conclusion

A fibrous bridge can adequately transmit sound to the stapes, resulting in a near normal auditory function. In such cases ossicular reconstruction may be unnecessary.

## Conflicts of interest

The authors declare no conflicts of interest.
